# Antimicrobial Activity of *Syzygium aromaticum* Essential Oil in Human Health Treatment

**DOI:** 10.3390/molecules29050999

**Published:** 2024-02-25

**Authors:** Valentina Maggini, Giulia Semenzato, Eugenia Gallo, Alessia Nunziata, Renato Fani, Fabio Firenzuoli

**Affiliations:** 1 Center for Integrative Medicine, Careggi University Hospital, University of Florence, 50124 Florence, Italyeugenia.gallo@unifi.it (E.G.); alessia.nunziata@edu.unifi.it (A.N.); 2 Department of Biology, University of Florence, Via Madonna del Piano 6, Sesto Fiorentino, 50019 Florence, Italy; giulia.semenzato@unifi.it

**Keywords:** *Syzygium aromaticum*, essential oil, eugenol, antimicrobial activity, human infections, endophytes

## Abstract

The use of natural compounds to prevent and treat infective diseases is increasing its importance, especially in the case of multidrug-resistant (MDR) microorganisms-mediated infections. The drug resistance phenomenon is today a global problem, so it is important to have available substances able to counteract MDR infections. *Syzygium aromaticum* (L.) Merr. & L.M. Perry (commonly called clove) is a spice characterized by several biological properties. Clove essential oil (EO) consists of numerous active molecules, being eugenol as the principal component; however, other compounds that synergize with each other are responsible for the biological properties of the EO. *S. aromaticum* is traditionally used for bowel and stomach disorders, cold and flu, oral hygiene, tooth decay, and for its analgesic action. Its EO has shown antioxidant, antimicrobial, anti-inflammatory, neuro-protective, anti-stress, anticancer, and anti-nociceptive activities. This review aims to investigate the role of *E. S. aromaticum* EO in the counteraction of MDR microorganisms responsible for human disorders, diseases, or infections, such as *Escherichia coli*, *Pseudomonas aeruginosa*, *Staphylococcus aureus*, *Salmonella typhi*, *Candida albicans*, *Giardia lamblia*, *Streptococcus mutans*, *Porphyromonas gingivalis*, and *Klebsiella pneumoniae*. This study might orient clinical researchers on future therapeutic uses of *S. aromaticum* EO in the prevention and treatment of infectious diseases.

## 1. Introduction

Essential oils (EOs) are mixtures of volatile and aromatics compounds, mostly represented by terpenes (monoterpenes and sesquiterpenes) and, in smaller quantities, aldehydes, alcohols, ethers, and phenols. These molecules are poorly soluble in water, and they can be extracted by different techniques [1]. EO compounds are responsible for various biological functions, such as the antimicrobial activity against Gram-negative and Gram-positive bacteria, yeasts, and fungi [2,3].

Over the past few years, there has been an increase in human infections that have become lethal due to the spread of multidrug-resistant (MDR) pathogens [4]. The antibiotic resistance phenomenon represents a huge problem in healthcare leading to the necessity to discover and use new molecules, such as EOs [5].

Since EOs are composed of multiple compounds, the onset of resistance to these molecules is largely reduced [5]. Indeed, EOs can act on different cellular targets: the first structure to be affected is the cell membrane, as the lipophilic substances of the EOs can modify its correct functioning; moreover, they can also interfere with enzymes and proteins function and with fatty acids metabolism [5].

### 1.1. Syzygium Aromaticum: Botanical Aspects

**Figure 1:**
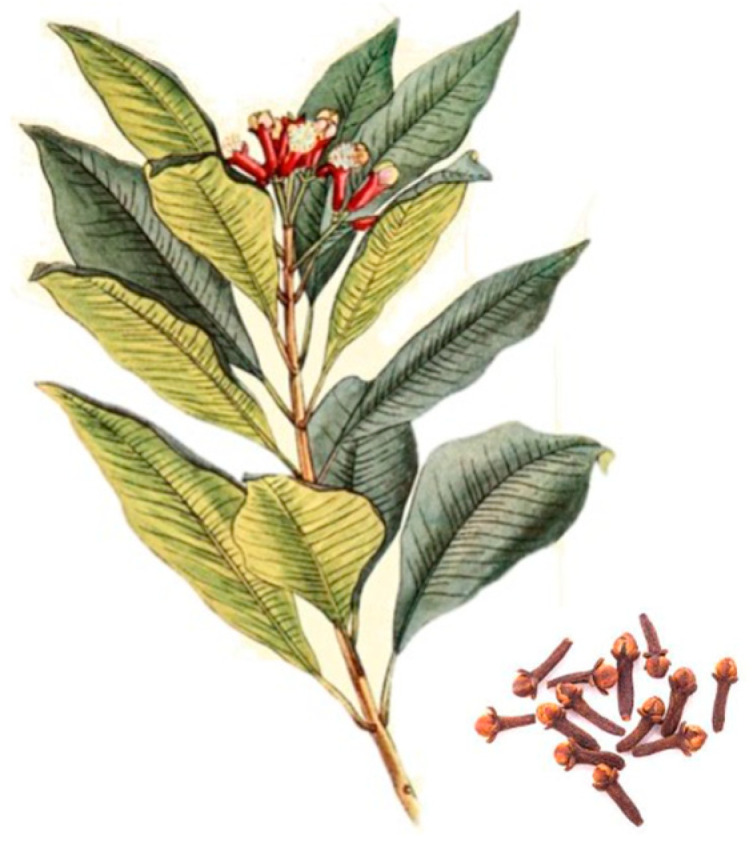
Representation of *S. aromaticum*.

*Syzygium aromaticum* (L.) Merr. and L.M. Perry or *Eugenia caryophyllata* Thunb., commonly referred to as clove, is a tree belonging to the Myrtaceae family and is a medicinal plant promising for its antimicrobial use. The plant is native to Indonesia, in particular from Maluku islands, and requires a warm and humid climate. The evergreen tree can reach a height of 12–15 m, and it is characterized by ovate-lanceolate leaves, flowers with 4 red sepals and 4 white-pink petals, and berries as fruit (Figure 1) [6,7].

After 4 years of cultivation, flower buds begin to form (their shape resembles that of a nail), and they are typically harvested before flowering, during maturation. Using natural phytohormones, it is possible to produce a precocious maturation [6]. 

The buds represent the plant compartment with the highest content of EO, but it can also be obtained from leaf distillation [7]. Furthermore, clove represents one of the most important sources of phenolic compounds, such as kaempferol and quercetin, caffeic acid, ellagic acid, and ferulic acid [6].

### 1.2. Chemical Composition of S. aromaticum EO

Clove EO is composed of several compounds. The main one is eugenol, representing 50% of its composition. Eugenyl acetate, β-caryophyllene, and α-humulene are other EO components, typically present in lesser quantities [8,9]. Different factors influence the EO composition, including the distillation method and/or plant variety [10]. 

**Figure 2:**
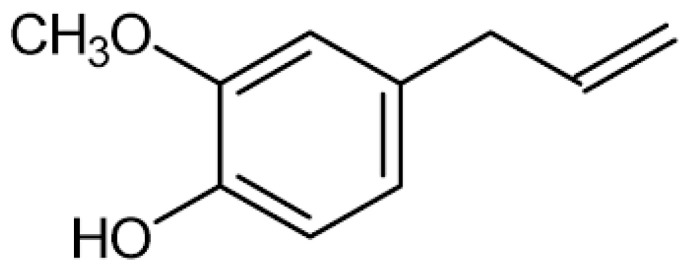
Chemical structure of eugenol [11].

Eugenol is a phenylpropanoid and represents the most bioactive volatile molecule of clove EO, characterized by an intense taste and smell (Figure 2) [11]. Its solubility in water is low, while it is high in organic solvents [12]. When administered orally, eugenol is well absorbed and easily reaches the bloodstream. In 24 h, it is completely excreted in the urine as metabolite conjugates, mostly eugenol-glucuronide and sulfate. Only 0.1% of the administered dose is excreted without conjugation [13]. The toxicity of eugenol is due to its prooxidant activity and its binding with lysine residues, which determines protein degradation [13]. However, both *S. aromaticum* EO and eugenol are considered safe by the Food and Drug Administration and, in accordance with the World Health Organization, the recommended dose is 2.5 mg/kg [9].

*S. aromaticum* EO can be obtained by different conventional methods, but also from innovative techniques. The most common method is distillation: using boiling water or steam, all volatile components are distilled from the plant matter; being insoluble in water, EO separates from the rest, resulting in the generation of two easily separable phases. Despite its simplicity, this method has some downsides, as it can be responsible for the degradation of some EO components [8]. Ultrasonic-assisted extraction (UAE), microwave-assisted extraction (MAE), and supercritical fluid extraction (SFE) represent some of the innovative techniques that guarantee a faster extraction with a lower use of energy, if compared to conventional methods. In these cases, the volatile components are obtained from the plant material after cell wall degradation. When using MAE, the content of eugenol in *S. aromaticum* EO is high and preserves its antioxidant and antimicrobial properties [8]. UAE uses ultrasonic waves that can be applied both directly to the plant samples and indirectly to its container [8]. In the SFE, a solvent, generally carbon dioxide in its supercritical state is used. The addition of co-solvents such as ethanol or water may change the features of the supercritical fluid thus facilitating the extraction of specific compounds of interest. This method eliminates possible pollutants such as pesticides and toxic substances. The comparison of *S. aromaticum* EOs obtained using these three different methods highlighted the presence of the same main compounds, although in different concentrations [8].

## 2. Biological Activities of *S. aromaticum*

**Figure 3:**
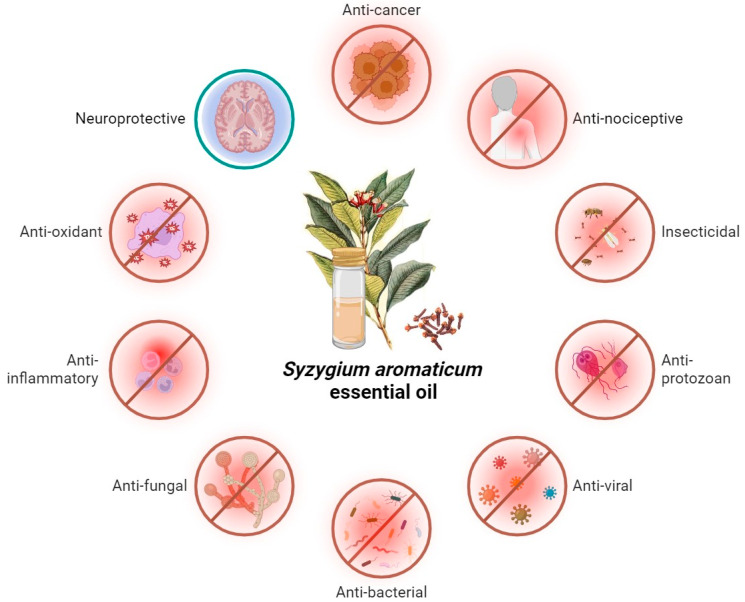
Schematic representation of the biological activities of *S. aromaticum* essential oil. (Created with BioRender.com, accessed on 8 January 2024).

*S. aromaticum* is characterized by many pharmacologic activities, schematized in Figure 3, which can allow the maintenance of human health [9]. 

It performs an antioxidant action, inhibiting lipid peroxidation and the formation of reactive oxygen species (ROS). Indeed, many human diseases, such as cancer, arthritis, and diabetes, are characterized by the presence of large quantities of free radicals, and it is known that the intake of plants, fruits, and vegetables rich in flavonoids, polyphenols, and anthocyanins can be beneficial, due to their anti-scavenging action. In this context, eugenol can exert significant antioxidant activity because of the presence of an allyl group [12]. 

The anti-inflammatory activity of eugenol is also well-known; it consists of the inhibition of cyclooxygenase-2 (COX-2), inflammatory cytokines, prostaglandin synthesis, and TNF-α, and NF-KB activation [12]. 

Regarding neuroprotective and anti-stress activities, it has been demonstrated that eugenol can reduce the levels of amyloid-β peptide, inhibit 5-lipoxygenase, and avoid the reduction in dopamine contents [12]. 

Clove EO and eugenol are also used for their analgesic effect in conditions of general sickness, headaches, or oral diseases. The analgesic activity is due to the interaction with the cholinergic and the opioid systems, and to the inhibition of voltage-dependent sodium channels and activation of transient receptor potential cation channel V1 (TRPV1), in the same way as local anesthetics [8]. 

An additional biological activity of clove EO is the anticancer activity since it can induce cell death against different tumor types, such as colon, lungs, prostate, and pancreas cancers [8]. It inhibits prostaglandin E2 production, the DNA oxidation process and it also suppresses the expression of the COX-2 gene in human colon cancer cell lines [12]. It also decreases chemotherapy side effects, such as vomiting, loss of appetite and weight, and nausea [8]. 

*S. aromaticum* EO can also induce an anti-nociceptive activity, due to COX-2 inhibition, activation of opioid and cholinergic system receptors, and the modulation of gamma-aminobutyric acid (GABA) receptors [8]. 

Finally, clove EO can be used for its insecticidal activity against numerous insects and parasites such as fleas, aphids, mites, ants, and mosquitoes, without any damage to human health and the environment which are often associated with the use of common insecticides [8].

This review will focus on the antimicrobial activity of *S. aromaticum* EO, i.e., its action against bacteria, viruses, and fungi.

### 2.1. Antimicrobial Activity of S. aromaticum EO

*S. aromaticum* EO exhibits an important antimicrobial activity against several microorganisms (Table 1). Because of their lipophilic characteristics, EOs can interfere with the membrane structure causing the loss of its stability and cellular content leaking, thus resulting in cell death. *S. aromaticum* EO can interfere with the growth of both Gram-negative bacteria, such as *Salmonella typhimurium*, *Escherichia coli*, *Klebsiella pneumoniae*, *Pseudomonas aeruginosa,* and *Agrobacterium* spp., and Gram-positive ones, including *Streptococcus* spp. and *Staphylococcus aureus*. It is also effective against yeasts, such as *Candida albicans*, *Penicillium*, and *Aspergillus* spp. [8]. Different studies showed that eugenol and its derivatives have also antiviral activity against different forms of flu, herpes simplex virus, and even against the first step of HIV-1 infection, by reducing virus replication [8]. 

#### 2.1.1. Antibacterial Activity

Some evidence of the antibacterial activity of *S. aromaticum* EO came from the microbiology Laboratory of the Federal University of Maranhão. Teles and colleagues (2021) determined *S. aromaticum* EO and eugenol antimicrobial activity via the disc-diffusion test on bacterial strains belonging to the species *S. aureus* (Gram-positive) and *E. coli* e *P. aeruginosa* (Gram-negative) [14]. After 24 h of incubation, both *S. aromaticum* EO and eugenol induced a consistent inhibitory activity against *S. aureus* [14]. Concerning the Minimal Inhibitory Concentration (MIC), clove EO was more effective against *S. aureus* (MIC = 50 μg/mL) than eugenol (MIC = 250 μg/mL) [14]. Even though it has been reported that *S. aromaticum* EO’s antimicrobial activity is largely due to the presence of eugenol, other phenolic compounds might be responsible for this activity [3]. The MIC values obtained in that study highlighted that the phytocomplex, rather than the single compound, is responsible for such activity [14]. Gram-negative species *E. coli* and *P. aeruginosa* were less inhibited by clove EO, probably because of the presence of the external membrane that represents an additional barrier to the introgression of EO components, if compared to Gram-positive bacteria [5].

Overall, the antimicrobial activity of an EO can be influenced by different factors: the characteristics of the target microorganism(s), temperature, pH, concentration of antimicrobial substances, and/or the presence of organic matter. Hence, the results obtained in vitro should not be compared to the in vivo applications of natural compounds since, in the latter case, the antimicrobial activity could be reduced or not manifest at all [2]. Clove EO has been used for the evaluation of the influence of these factors on its antimicrobial effect against *E. coli*, *S. aureus*, and *P. aeruginosa* [2,15]. At first, it was observed that the antimicrobial activity of *S. aromaticum* EO was twenty times higher at the temperature of 37 °C [2]. The fluidity of the membrane lipid layer is influenced by the temperature; thus, at higher temperatures, the cell membrane function is compromised, and the permeability increases, with the consequent rise of cell susceptibility to the antimicrobial compound(s) [16]. Concerning the presence of organic material, the bactericidal activity of clove EO was preserved, even though reduced, highlighting its potential as an antimicrobial agent for external use, for example in dentistry or for the treatment of skin issues [2].

**Table 1:** Biological activities of *S. aromaticum*.

Year	Product from *S. aromaticum*	Biological Activities	Mechanisms of Action	Reference
2021	Essential oil hydrodistilled from buds (São Luís, Maranhão, Brazil)	antioxidant, antibacterial, antitrypanosomal	membrane protein inactivation by eugenol hydroxyl group, cell membrane ruptures, modulation of NO production, downregulation of NF-kB and AP-1	[14]
2018	Solid lipid nanoparticles containing essential oil, water-distilled from buds (Karaj city, Alborz province, Iran)	antibacterial, antifungal	interaction with microbial cell membrane	[17]
2021	Essential oil-encapsulated nanofiber formulation (Sigma Aldrich, Darmstadt, Germany)	antibacterial, wound healing	interaction with microbial cell membrane, antioxidant effect	[18]
2020	Essential oil (Fagron, Wichita, KS, USA, L150102014)	antibacterial	interaction with microbial cell membrane, synergy with antibiotics	[19]
2017	Essential oil (Apple Flavor & Fragrance Group Co. Ltd., Shanghai, China)	antibacterial	modulation of cell membrane permeability, biofilm inhibition	[20]
2010	Essential oil, hydro-distilled from buds	antibacterial, antifungal, anticarcinogenic	interaction with microbial cell membrane, eugenol cytotoxicity	[21]
2007	Essential oil, hydro-distilled from buds (Monastir, Tunisia)	antioxidant, antifungal, antibiofilm	free radical quenching, interaction with microbial cell membrane,	[22]
2005	Essential oil	antifungal	interaction with microbial cell membrane	[23]
2018	Vaginal gel containing plant extracts	antibacterial, antifungal, antibiofilm	interaction with microbial cell membrane	[24]
2011	Essential oil, obtained from buds (Segredo da Planta’, Paio Pires, Portugal)	anti-*Giardia*	Loss of cell polarity, adherence inhibition	[25]

#### 2.1.2. Nanofibers and Nanoparticle Formulations

Nanotechnology includes various innovative systems that consist of the inclusion of antimicrobial substances in chemical matrices to improve their effectiveness [17]. In the context of multidrug resistance, it is known that the infections caused by MDR human pathogens are more difficult to eradicate with conventional antibiotics, making necessary the utilization of new antimicrobial compounds and/or new formulations. Nanofibers loaded with clove EO recently found application in wound healing processes. The formulation composed of *S. aromaticum* EO, chitosan, and polyethylene oxide exerted a strong antibacterial effect against *S. aureus*, *E. coli*, *P. fluorescens*, and *B. subtilis* [18]. When used in disc-diffusion tests, the EO formulation determined the appearance of diffuse and pronounced inhibition halos, if compared to the nanofibers in the absence of the EO [18]. The same EO-loaded nanofibers have been tested in vivo on Sprague Dawley rats to evaluate wound healing. Circular wounds were created on the back of the animals; afterward, rats were separated into 4 groups, which received (i) no treatment, (ii) application of empty nanofibers, (iii) applications of EO-loaded nanofibers, and (iv) treatment with a commercial product. Nanofibers loaded with clove EO exerted a similar healing ability as the commercial product, thus confirming their potential applications for this purpose [18].

Even though *S. aromaticum* EO is an effective antimicrobial mixture, its activity can be limited because of its hydrophobicity and high volatility. For these reasons, nanoparticle formulations can represent an alternative valid system to overcome these limits and improve clove EO antibiotic activity [17]. One of the advantages of using nanoparticles is that they are characterized by low cytotoxicity, and they allow the increase of EO stability, while controlling their pharmacokinetics [17]. The antibacterial effect of clove EO alone and EO-loaded nanoparticles was checked and compared using *Salmonella typhi*, *P. aeruginosa*, *S. aureus*, and *Candida albicans* as target strains, through the determination of the MIC. Also in this case, the antimicrobial activity of EO-loaded nanoparticles was higher than that of the EO alone; it has been suggested that the nano-formulation interacts better with the microbial cell membrane and stabilizes the EO, resulting in a more pronounced antimicrobial activity, mostly against Gram-negative bacteria and fungi [17]. On the other hand, the formulations had lower efficacy against Gram-positive bacteria when compared to the EO alone [17].

#### 2.1.3. Nosocomial Pathogens

The World Health Organization has released a list of microorganisms, including *Acinetobacter baumannii* and *Klebsiella pneumoniae*, for which the identification of new substances capable of combating them is of great importance and absolute urgency [19]. 

Colistin is one of the few drugs still used against resistant *A. baumannii* and *K. pneumoniae* strains. Clove EO was tested against colistin-susceptible strains of *A. baumannii* and *K. pneumoniae*, in combination with the antibiotic, via the disc-diffusion test. Data obtained demonstrated the existence of a synergy between *S. aromaticum* EO and colistin against both bacterial strains, a synergy that was not observed with other types of antibiotics, suggesting a specific interaction between the clove EO and colistin [19]. Because of this synergy, checkerboard essays were set up also for strains resistant to the antibiotic, highlighting that clove EO was able to decrease the antibiotic MIC. This effect was attributed to the hydrophobic nature of eugenol, which disrupts cell membrane; in addition to this, also colistin, having an amphipathic nature, can interact with Gram-negative external membrane. Since the two molecules act similarly, the resulting membrane permeability alteration induces cell death [19]. The administration of colistin, when combined with other antimicrobial substances that it synergizes with, allows for the retention of its biological activity at reduced concentrations [19]. Since colistin exhibits a certain degree of toxicity in humans, in a dose-dependent way, the lower the dose, the fewer the side effects.

Further studies are needed, but it can be concluded that the combination of *S. aromaticum* EO and colistin represents a good example of a combination of substances with antimicrobial activity against resistant strains.

#### 2.1.4. *S. aromaticum* EO in the Treatment of Oral Cavity Diseases

*S. aromaticum* EO has been used in different medical devices, cosmetic products, and food. Particularly, eugenol has a strong activity against various bacteria colonizing the oral cavity such as the Gram-negative anaerobic *Porphyromonas gingivalis*, which is responsible for periodontal disease. This disease, where the gingiva is degraded until the teeth fall out, is conventionally treated with antibiotics to reduce the bacteria layer. However, these periodontal pathogens form a biofilm in the oral cavity thus becoming resistant to the action of many antimicrobial agents and elude the host immune system cells. In this scenario, it appears necessary to identify new suitable substances able to remove oral cavity biofilms, without the side effects often associated with the usage of conventional antibiotics [20].

Zhang and colleagues (2017) tested the antibacterial activity of both eugenol and EO distilled from clove leaves against *P. gingivalis* [20]. Both MIC and minimum bactericidal concentration (MBC) were obtained through the broth microdilution essay, using tinidazole as a positive control [20]. A strong antibacterial effect against *P. gingivalis* was exerted by both *S. aromaticum* EO and eugenol [20]. The antibiotic-kill curves obtained highlighted that both eugenol and clove EO reduced the quantity of live bacterial cells proportionally to time; after 4 h, most of the cells were killed [21]. The mechanisms of action were attributed to changes in bacterial cell membrane permeability, as suggested by the observed release of intracellular material, like protein and nucleic acids, after the 4 h treatment with eugenol. Moreover, eugenol was able to interfere with the initial steps of bacterial biofilm formation. Instead, its effect on existing biofilm was less pronounced, even though detectable [20]. 

Among the bacteria responsible for the onset of tooth decay, *Streptococcus mutans* is considered the main cariogenic agent. It can survive in an environment at low pH, which is very likely due to the bacterial carbohydrates metabolism that is responsible for the production of acids. The treatment of *S. mutans* infection includes the use of antibiotics, which may allow to eradicate the bacterial infection; however, antibiotics may also be responsible for oral and/or intestinal microbiota alterations [21]. Dental caries can be also determined by yeasts, like *Candida* spp., which mainly affect the teeth root rather than the entire surface, like bacteria [21]. The antimicrobial activity of *S. aromaticum* against some human pathogenic bacteria and yeasts isolated from patients with caries and tooth decay was evaluated in the study of Kouidhi et al. (2010). The disc-diffusion test revealed that clove EO, even at low concentrations, possessed an excellent antibacterial activity against oral streptococci, including cariogenic bacteria, as well as a worthy antifungal activity [21]. 

Based on these results, it has been suggested that *S. aromaticum* EO can have promising employment in dental hygiene products to prevent chronic inflammation of the gums and bacterial and fungal infections [20].

#### 2.1.5. Antifungal Activity against Different Species of Candida

Candidiasis refers to a fungal infection caused by *Candida* species, predominantly *Candida albicans*. It is a prevalent opportunistic infection affecting various parts of the body, commonly the skin, mucous membranes, and gastrointestinal tract [26]. *Candida* is part of the normal human microbiota, but an overgrowth due to weakened immunity, antibiotic use, hormonal changes, or underlying medical conditions can lead to candidiasis. The rising incidence of candidiasis is of great concern because of its association with antibiotic resistance, making its treatment challenging [26]. Addressing this issue involves understanding the risk factors, improving diagnostics, promoting preventive measures, and developing novel antifungal agents [26]. 

The antifungal activity of *S. aromaticum* EO against *Candida* strains isolated from hospital patients with candidiasis was observed by Chaieb et al. (2007) using the disc-diffusion method [22]. *S. aromaticum* EO was effective against different human pathogenic yeasts and its inhibitory activity was attributed once again to eugenol which can perturb the integrity of cell membranes [22]. 

Some species of *Candida*, together with dermatophyte fungi like *Epidermophyton flocosum*, *Tricophyton rubrum*, or *Tricophyton mentagrophytes* var. *interdigitale*, are responsible for some forms of onychomycosis, an infection that involves feet nails [23]. The effect of *S. aromaticum* EO and eugenol has been evaluated against different microbial strains like *C. albicans*, *C. tropicalis*, *C. krusei*, *T*. *rubrum*, and *T. mentagrophytes*, comparing their activity with common antifungals. The disc-diffusion test and the MIC obtained showed that both the EO and eugenol were effective against all microorganisms [23]. 

Since the increasing need for new substances with low adverse effects able to counteract fungal infections, different formulations of *S. aromaticum* EO and eugenol can represent a valid alternative to conventional antifungals to be used in the treatment of candidiasis and onychomycosis [23]. 

#### 2.1.6. Bacterial Vaginosis and Vulvovaginal Candidiasis

Vaginitis in premenopausal women consists of an inflammatory condition caused by microbial infections, such as bacterial vaginosis (BV) or vulvovaginal candidiasis (VVC). In most cases, these conditions resolve spontaneously, while sometimes the symptoms persist or resurface after the treatments. Approximately 75% of women have experienced in their lives symptomatic VVC, often characterized by recurrent episodes where the symptoms reoccur after a few weeks or months [24]. However, the most common vaginitis trigger is BV, characterized by the absence of hydrogen peroxide-producing lactobacilli, normal colonizers of the vaginal mucosa, together with the increase in the number of anaerobic lactobacilli. The bacterial vaginosis is generally caused by *Gardnerella vaginalis*, *Atopobium vaginae*, and some Bacteroides species [24]. BV and VVC are treated with oral or intravaginal antimicrobials. However, these drugs are not able to restore the microflora balance, so they cannot provide long-term protection. Moreover, the emergence of multidrug-resistant strains can make conventional drugs inefficient, so new therapeutic approaches are needed [24]. 

The goal of the non-interventional, observational, multicenter, open-label study conducted by Murina and colleagues (2018) was to evaluate the efficacy of a vaginal gel containing *S. aromaticum* and *Thymus vulgaris* EOs, in combination with two lactobacilli strains, formulated in slow-release vaginal capsules [24]. In this study, which involved women of childbearing age with BV and VVC symptoms, the formulation was used as the only therapy. A significant improvement in vaginal burning, itching, and discharge production, occurred and the microbiological evaluation was found to be normal in 80% of the cases. The therapy has been well tolerated and no one abandoned the study. A high percentage (80%) of women showed clinical and microbiological healing and the efficacy of treatment was the same for both disorders [24]. Consequently, *S. aromaticum* and *T. vulgaris* EOs appeared as powerful mixtures with antibacterial and antifungal activities that synergize with each other to inhibit the growth of microorganisms by producing alterations in the structure of the microbial membrane and lowering the pH, restoring the lactobacilli microflora [24]. 

In conclusion, the results encourage the utilization of such vaginal capsules for BV and VVC treatment, also in the case of recurrent episodes [24]. 

#### 2.1.7. Anti-Giardia Activity

Several diseases and intestinal infections affecting both humans and animals worldwide are caused by *Giardia lamblia.* It is a flagellated protozoan characterized by a cell cycle consisting of a growth phase and a dormant phase, i.e., the trophozoites and cysts, that allow the microorganism to adapt and survive in different environments and under different environmental conditions. This infection is very dangerous for children and elderly people because it is responsible for severe diarrhea and nutrient malabsorption, representing a relevant risk for people with immune deficiency. Particularly, travelers are the most exposed subject to *G. lamblia*, as the infection may occur through contact with contaminated food and water [25]. 

The presence of resistant strains and the scarce efficacy of available drugs have led to the necessity to obtain new plant-derived compounds with anti-*Giardia* activity. In this context, *S. aromaticum* EO may represent a potentially effective candidate to overcome this intestinal problem [25]. The activity of clove EO and eugenol against *G. lamblia* has been evaluated through the growth inhibition assay. The antimicrobial agents were incubated with the trophozoites, which were then observed under the optical microscope: both EO and isolated eugenol inhibited *G. lamblia* growth in a concentration-dependent way [25]. The trophozoites’ adhesion capacity was also evaluated. Since the adherence of this pathogen to intestinal cells is a determinant factor for its infection, preventing adherence or the detachment of trophozoites from intestinal walls are fundamental steps to allow the elimination of protozoans from the organism. Clove EO influenced trophozoites’ vitality and their adherence causing cell lysis. Instead, eugenol did not determine cell death but inhibited the adherence of the trophozoites. It can be concluded that the EO includes other components that are responsible for trophozoites’ death. The loss of adherence was determined by structural changes of trophozoites’ shape and reabsorption of flagella, caused by the action of clove EO components eugenol [25]. 

Other investigations and in vivo studies are necessary, but these in vitro assays showed that *S. aromaticum* EO and eugenol can inhibit protozoan development, its structure, and vitality, so they can be considered as effective compounds in the treatment of *Giardia* infections [25].

## 3. Conclusions

This review highlighted that clove EO has a major antimicrobial potential against different microbial strains and presented the possible applications of this phytocomplex in the treatment of various diseases and infections. The experimental results here presented showed positive outcomes and can be considered as a starting point for further studies. However, the studies reviewed in this paper were mainly conducted in vitro; the real efficacy of *S. aromaticum* EO should be taken into consideration only after careful in vivo analyses. An interesting outcome is represented by the synergy between the EO and/or its components with other antimicrobial compounds, which could help in facing the global issue of drug resistance, reducing at the same time the negative effects of commonly used medicines. New formulations like nanoparticles or nanofibers represent an innovative way to improve the effectiveness and stability of EOs, as well as the combination of EOs with microorganisms, like lactobacilli, to counteract infections and restore the balance of the human microbiota. The most critical aspect remains the identification of the right combinations to obtain an antimicrobial effect on a specific pathogenic target. This is the case of the observed synergy between clove EO and colistin for nosocomial infection treatment. 

Considering the urgent worldwide problem of antibiotic resistance, natural substances, such as the EO of *S. aromaticum*, represent the most promising way for innovative antimicrobial formulations.

## 4. Future Perspectives

To fully harness the pharmacological potential of plants and their EOs, it is essential to consider medicinal aromatic plants as complex and dynamic systems. Just like all other macro-organisms, medicinal plants possess a microbiota referred to as the phytobiome. This encompasses microorganisms capable of colonizing the plant’s internal tissues (referred to as endophytes), not causing harm or signs of infection to their host [27]. 

Most bacterial endophytes originate from the rhizosphere, the soil portion surrounding the plant roots, attracted by root secretions, and then reach various plant parts through the xylem vascular system [28]. The plant microbiota includes commensals, not affecting plant growth but using their host’s metabolites for survival, as well as beneficial microorganisms (Plant Growth Promoter Bacteria, PGPB), which receive nourishment and protection from their host while aiding in plant growth and defense mechanisms [29].

Research on bacterial endophytes of clove is quite limited and should be deepened. It is now clear that the understanding of the multiple interactions between medicinal plants and endophytes could revolutionize plant biology and pave the way for modulating and enhancing plants phytochemical production, and/or potentially obtaining biologically active molecules directly from plant-isolated endophytes [30,31]. 

**Figure 4:**
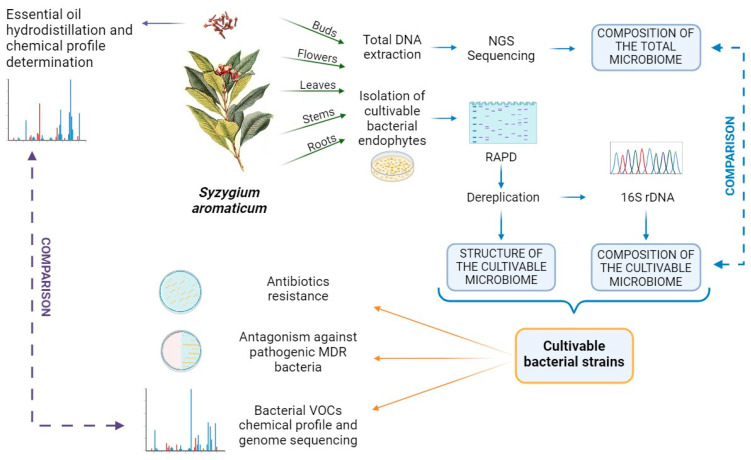
Experimental workflow for the molecular and phenotypic characterization of bacterial endophytes associated with *S. aromaticum*. The essential oil is obtained from the buds (and/or the leaves) of the plant. After surface sterilization, small amounts of each anatomical part are used for both total DNA extraction and cultivable bacterial endophytes’ isolation. The DNA is sequenced to obtain the composition of the total (cultivable and non-cultivable) microbiota, while the culturable endophytic strains are subjected to both molecular and phenotypic analyses. Random Amplified Polymorphic DNA (RAPD) analysis and the 16S rRNA gene sequencing provide information on the structure and composition of each community, highlighting the degree of biodiversity and the possible microbiome divergences among different microenvironments. The collection of cultivable strains allows the phenotypic characterization of endophytes. It is possible to explore the levels of antibiotic resistance of each community and the ability of endophytes to interfere with the growth of MDR pathogens through the production of antibacterial molecules, mimicking the antimicrobial potential of the essential oil. The characterization of the volatile organic compounds (VOCs) produced by the strain could shed light on endophytes’ potential to produce similar, if not the same antibacterial VOCs and so contribute to the plant’s essential oil composition. (Created with BioRender.com, accessed on 8 January 2024).

Manipulable endophytic bacteria might represent a sustainable source of novel natural compounds. Indeed, the plant’s biological activities and the ability of endophytes to stimulate plant metabolism may be interlinked, and it prompts the question of whether the therapeutic properties of essential oils rely on the presence of molecules having a bacterial origin (Figure 4). As an example, extracts obtained from culturable endophytic bacteria from clove leaves, screened for their antioxidant ability, revealed endophytes’ potential as antioxidant producers, mimicking eugenol properties [32,33]. On the contrary, scientific works addressing clove endophytes’ antibacterial activity are still lacking. Recent studies on bacterial endophytes isolated from medicinal plants, such as *Lavandula angustifolia*, *Echinacea purpurea*, and *Origanum* spp., evidenced that bacterial endophytes have the potential to produce diffusible and volatile antimicrobial substances inhibiting the growth of bacterial human pathogens [34,35]. The purified active molecules from potent endophytes could undergo in vivo testing for their bioactivity, potentially matching the therapeutic effects of medicinal plants [36]. Moreover, the phenotypic and genotypic characterization of the antimicrobials-producing strains could lead to an easier and optimized antimicrobial compound(s) production [37,38]. Further experimental approaches, such as evaluating bacterial volatile organic compounds (VOCs) alongside essential oil compositions, hint at endophytic involvement in synthesizing essential oil components [35]. Endophytes demonstrated the capacity to stimulate the host plant’s metabolism. Studies on sterile plants inoculated with isolated endophytes displayed changes in secondary metabolite profiles, suggesting an endophytic influence on the plant’s secondary metabolism [39]. These preliminary but promising results highlight the biotechnological potential of endophytic bacteria from medicinal plants, underscoring the importance of exploring bacterial phytobiomes as potential weapons against multidrug-resistant pathogens infections.

## Data Availability

The data presented in this study are available in article.
